# Correspondence on international consensus on natural-orifice specimen-extraction surgery (NOSES) for colorectal cancer

**DOI:** 10.1093/gastro/goaa055

**Published:** 2020-11-03

**Authors:** Joseph W Nunoo-Mensah, Mariam Rizk

**Affiliations:** Department of Colorectal Surgery, King’s College Hospital Foundation NHS Trust, London, SE5 9AE, UK

Natural-orifice specimen-extraction surgery (NOSES) for colorectal disease is not a new surgical procedure, although its lack of penetration into common colorectal surgical practice may make it a relatively new procedure. Stewart *et al.* [1] were among the first to report the extraction of a colectomy specimen through the vagina in 1991 and, shortly thereafter, Franklin *et al.* [2] published the first report of partial colectomy with natural-orifice specimen extraction (NOSE) via the anus. Since then, there have been several publications on the extraction of both malignant and benign colonic diseases from the caecum to the distal rectum through natural-orifice extraction sites. Unlike procedures such as laparoscopic cholecystectomy, which was introduced in 1985 and rapidly diffused into the surgical community over a 2- to 3-year period, the dispersion of NOSES has not been equivocal [3]. As there are several reasons why some new surgical innovations may be taken up more quickly than others, the adoption curves of new procedures can take many forms. However, the tipping point that describes the onset of the peak rate of diffusion of the new technology usually occurs after the first 10%–20% of users have adopted it [3]. From a global perspective, the adoptions curve for NOSES has not obtained a peak rate of diffusion and appears to have arrested in the developmental and explorative phase of innovation [3].

The recent publication by Guan *et al.* [4] from the International Alliance of NOSES provides a succinct description of the classifications and indications for NOSES procedures. Strategies in reducing the risk of bacterial contamination of the peritoneal cavity and oncological safety when extracting the specimen are discussed. However, in order to properly evaluate NOSES, we need to ask the following questions: (i) Does it make sense? (ii) Are there any significant short-term benefits? (iii) Are there any adverse complications? (iv) Are there any long-term oncological implications?

Surgery is generally very slow to scrutinize the rapid progression of new surgical innovations until Level-1 evidence such as randomized control trials (RCTs) have shown them to be effective. However, it is challenging to evaluate a new surgical procedure in an RCT due to many potential practical problems: recruiting patients may be difficult, as they may refuse to be randomized; measuring appropriate outcomes may require years of follow-up; there may be differences in the surgical skills of the techniques being analysed; therefore, analysis should take account of how experienced each surgeon is in performing the new operation. Ideally, randomization should begin as soon as it is feasible, as this would enable the researchers to monitor the learning curve. The latter was emphasized in the recent ROLARR randomized clinical trial, which compared the effect of robotic vs laparoscopic surgery for rectal cancer. The median laparoscopic cases performed in the laparoscopic arm was 91 vs 50 in the robotic arm and, despite a recruitment of 471 patients, there was no difference in the primary endpoints of conversion to open laparotomy and positive rate of circumferential resection margin. A subsequent publication exploring and adjusting for potential learning effects showed that the initial ROLARR analysis was confounded by the learning effect and that the estimated odds ratio of conversion in the robotic arm was significantly lower after ∼70 cases were performed when compared with the laparoscopic arm with a median of 91 cases [5].

To date, there have been only three Level-1 publications on NOSES for colorectal procedures—two RCTs [6, 7] and one meta-analysis [8]. Wolthuis *et al.* [6] performed an RCT of 40 patients receiving either laparoscopic NOSE colectomy or conventional laparoscopic colectomy (CL) for left-sided colonic disease. In their study, the NOSE group had significantly less requirement for patient-controlled epidural analgesia, opioid and non-opioid, compared to the conventional-laparoscopic group. The post-operative pain scores were also significantly lower in the NOSE group. Interestingly, the inflammatory responses were significantly higher in patients undergoing NOSE colectomy and this group also had a significantly longer operative procedure and moderate increase in cost (median of 15 minutes and €494, respectively). Post-operative anorectal function, complications, and hospital stay were similar in the two groups. Leung *et al.* [7] described a novel technique of hybrid natural-orifice transluminal endoscopic surgery (NOTES) colectomy (HNC), which was effectively a NOSES procedure. The specimens were delivered through the anus using the transanal endoscopic operation device. During a 3-year period, the authors recruited 70 patients who were divided into two equal groups who underwent HNC and CL. The authors stated that the maximum-pain score during the first week was significantly lower in the HNC group. No patients in the HNC group developed wound infection, whereas four patients in the CL group did so (*P* = 0.005). There were no other statistical differences in the other standard parameters measured. Ma *et al.* [8] meta-analysis of NOSES vs laparoscopy for colorectal disease that included nine studies involving 837 patients supported the notion that laparoscopic resection with NOSE for colorectal disease can significantly reduce the duration of hospital stay, accelerate post-operative recovery with better cosmetic results, and, in particular, result in less post-operative pain and fewer complications. There appeared to be no difference in the disease-free survival (DFS), although the Log hazard ratios of only two of the studies were included for analysis. Although a meta-analysis is a Level-1 study, caution should be exercised in interpreting the results of this analysis, as eight of the studies included were non-randomized studies; this increases the risk of bias.

So, from a critical point of view, the concept of NOSES makes sense in the avoidance of incision-related morbidity as a goal of modern minimally invasive colorectal surgery. The approach also appears to offer significant short-term benefits with no immediate adverse complications. There is still the concern of the long-term oncological implications of NOSES, although, reassuringly, a large prospectively collected study of 844 patients (163 NOSE and 681 CL) who underwent curative surgery for rectal cancers showed a combined 5-year DFS rate for all stages of 89.3% in the NOSE group and 87.3% in the CL group (*P* = 0.639) [9]. There is no doubt that larger prospective RCTs are required but, for the reasons stated above, these are likely to be challenging. As with other recent surgical innovations such as transanal total mesorectal excision (taTME), establishing a large global registry of reported patients may be a secondary option to the early identification of complications and recording long-term outcomes. Although such registries rely on individual surgeons to meticulously, unbiasedly, and accurately record data.

Surgeons who typically perform laparoscopic-assisted colectomies would be faced with a steeper learning curve when adopting NOSES. Intracorporeal anastomosis is a prerequisite skill for those adopting NOSES. Furthermore, specimen extraction via the vagina requires a posterior colpotomy, which will be a new skill for most colorectal surgeons. These technical challenges are amplified by a lack of standardization of the technique. Guan *et al.* [4], in their consensus publications, together with a book published by Wang [10] standardize the NOSES procedure to encourage surgeons who may be faced with a steeper learning curve on how to adopt this procedure.

## Conflicts of interest

None declared. 

## Notes

Authors’ reply

Xu Guan and Xi-Shan Wang

Department of Colorectal Surgery, National Cancer Center/National Clinical Research Center for Cancer/Cancer Hospital, Chinese Academy of Medical Sciences and Peking Union Medical College, Beijing, P. R. China

Thanks so much for the valuable suggestions on the consensus. As suggested by the author, the adoptions curve for NOSES might not obtain a peak rate to spread from a global perspective. However, according to the data from China NOSES Database (CNDB), NOSES for colorectal cancer (CRC) has attracted widespread attention in China recently and >200 medical centers have begun to perform NOSES; the number of NOSES is still maintaining a steep upward trend in China. There are several reasons that have led to the fast progress of NOSES in China. First, laparoscopy has been widely used for the treatment of CRC, which has provided the basic support of equipment for the popularization of NOSES. Second, intra-abdominal digestive tract reconstruction, being considered as the key technical requirement for NOSES in CRC, has become much more feasible and safer with the advancement of laparoscopic technology. Third, the minimally invasive advantages of NOSES, including less pain and fewer wound complications, shorter hospital stay, and better cosmetic and psychological effects, allowed more surgeons to prefer it.

There has been a long-standing controversy regarding whether NOSES can influence the oncologic outcomes of CRC patients. Currently, there is no strong evidence from large prospective RCTs to provide acceptable short-term and long-term outcomes. This year, the China NOSES Alliance performed a large national registry study, which included >5,000 cases of NOSES on CRC, to further evaluate the short-term and long-term outcomes at a national level. The results from this national report have confirmed the perioperative safety and good long-term prognosis of NOSES, which will be published in the near future. I think this result will largely support the further promotion of NOSES.

As we know, many surgeons were initially against the application of the laparoscopic technique, especially for the treatment of cancer. However, laparoscopic techniques developed rapidly and became a regular treatment approach in just a few years. Therefore, we thought that, after the creation of any innovative surgical technique, it is necessary to experience it in clinical practice to explore the clinical feasibility. At the same time, high-quality clinical research can be performed to further confirm the safety of the surgical technology.

NOSES always puts forward high technical demands on tumor-free procedures in the treatment of CRC. Currently, numerous technical tips and experience regarding preventing tumor dissemination have been summarized in clinical practice, which have been widely recommended by more and more surgeons. Furthermore, the International Alliance of NOSES has done lots of work, including publishing books and consensus on NOSES, and holding workshops on NOSES for surgeons to learn and adopt this novel surgical procedure, which have been of great significance in the standardization and popularization of NOSES worldwide.

With the development and application of various equipment and platforms, surgical concepts and techniques will change greatly. Looking forward to the next 30–50 years, innovative minimally invasive techniques will be considered as a mainstream trend. If we cannot prevent the trend, then we should adapt to this new trend. Therefore, for the new surgical technique of NOSES, there is more work to be done to improve the theoretical system and technical details, thereby benefitting more patients and surgeons.
